# Exploring Analysis Approaches for Using the Dopamine Transporter Striatal Binding Ratio in Early‐ to Mid‐Stage Parkinson's Disease Modification Trials

**DOI:** 10.1002/mdc3.14191

**Published:** 2024-08-22

**Authors:** Nirosen Vijiaratnam, Christine Girges, Dilan Athauda, Alexa King, Grace Auld, Rachel McComish, Kashfia Chowdhury, Simon Skene, Kate Maclagan, Kallol Ray Chaudhuri, Vincenzo Libri, John Dickson, Thomas Foltynie

**Affiliations:** ^1^ Department of Clinical and Movement Neurosciences Institute of Neurology, University College London London United Kingdom; ^2^ Unit of Functional Neurosurgery, National Hospital for Neurology and Neurosurgery, Queen Square London United Kingdom; ^3^ The Francis Crick Institute London United Kingdom; ^4^ The Comprehensive Clinical Trials Unit, University College London London United Kingdom; ^5^ Surrey Clinical Trials Unit, University of Surrey Guildford United Kingdom; ^6^ Department of Clinical and Experimental Medicine University of Surrey Guildford United Kingdom; ^7^ Parkinson's Foundation International Centre of Excellence, King's College London London United Kingdom; ^8^ Leonard Wolfson Experimental Neurology Centre, National Hospital for Neurology and Neurosurgery Queen Square, London United Kingdom; ^9^ Institute of Neurology University College London London United Kingdom; ^10^ NIHR Clinical Research Facility, University College London Hospitals NHS Foundation Trust London United Kingdom; ^11^ Institute of Nuclear Medicine, University College London Hospitals NHS Trust London United Kingdom

**Keywords:** Parkinson's disease, presynaptic dopaminergic imaging, specific binding ratio, analysis approaches, disease modification

## Abstract

**Background:**

The dopamine transporter striatal binding ratio (DAT SBR) has been used as an outcome measure in Parkinson's disease (PD) trials of potential disease‐modifying therapies; however, both patient characteristics and analysis approach potentially complicate its interpretation.

**Objective:**

The aim was to explore how well DAT SBR reflects PD motor severity across different striatal subregions and the relationship to disease duration, and side of onset.

**Methods:**

DAT SBR for the anterior and posterior putamen and caudate in both hemispheres was obtained using validated automated quantitative software on baseline scans of 132 patients recruited for the Exenatide PD2 and PD3 trials. Associations between mean and lateralized SBR subregions (posterior and anterior putamen and caudate) and summed and lateralized motor characteristics were explored using regression analysis. Analyses were repeated considering disease duration and limiting analysis to the less‐affected hemisphere.

**Results:**

Lateralized bradykinesia was most consistently associated with the loss of DAT uptake in the contralateral anterior putamen. There was much higher variance in the posterior putamen, and in all regions in those with longer duration disease, although bradykinesia remained robustly associated with anterior putaminal DAT uptake even in longer‐duration patients. Restricting analyses to the less‐affected side did not usefully reduce the variance compared to the overall cohort.

**Conclusion:**

These data suggest that DAT SBR could be a useful biomarker in disease‐modifying trials, but a focus on anterior striatal subregions and incorporating disease duration into analyses may improve its utility.

Dopamine transporter single‐photon emission computed tomography (DAT‐SPECT) reflects nigrostriatal dopaminergic functional capacity in Parkinson's disease (PD).^1^ The specific binding ratio (SBR) is a quantitative index of DAT binding, which can reflect symptom severity resulting from dopaminergic impairment.^2^, ^3^, ^4^, ^5^ Striatal dopaminergic dysfunction typically begins in the posterior putamen in the premotor disease stage before gradually progressing in a nonlinear manner to involve anterior striatal subregions.^6^, ^7^ An analysis of the Parkinson's Progression Markers Initiative (PPMI) data showed a significant correlation between Movement Disorder Society Unified Parkinson's Disease Rating Scale (MDS‐UPDRS), Part 3, scores and DAT SBR using either the mean bilateral putamen score or the score of the putamen contralateral to the most clinically affected side at presentation. By year 4, the strength of the relationship between MDS‐UPDRS, Part 3, scores and DAT SBR is greatly diminished. Moreover, even in the early years, there are only weak correlations between the change in the MDS‐UPDRS, Part 3, scores over time and the change in DAT SBR, considering mean putamen, mean caudate, whole striatum, or contralateral putamen.^8^ This theoretically undermines the potential of DAT SBR as an objective, sensitive measure of disease progression that might be used in trials of potential disease‐modifying agents.

Part of the explanation of the lack of this relationship is that the MDS‐UPDRS, Part 3, does not capture only the dopaminergic elements of PD, and this is particularly so with disease progression due to extra‐striatal degeneration, for example, the development of axial signs of PD. Indeed, there have been previous explorations of the relationships between DAT SBR and specific clinical features of PD, indicating that bradykinesia is most consistently related to DAT SBR.^2^, ^3^, ^4^, ^5^, ^9^, ^10^ Additionally, the relationship between DAT SBR and clinical severity may be influenced by compensatory mechanisms,^11^ and long‐duration effects of dopaminergic replacement therapies,^9^, ^10^, ^12^, ^13^, ^14^ and the extent of these confounders may vary with advancing disease.

DAT SBR values may also be limited by floor effects, especially when considering the most affected striatum and/or if analyses include the posterior putamen, which is often almost completely lost even in early disease. There has therefore been interest in using the DAT SBR ipsilateral to clinical onset (ie, the least affected side), which may be less vulnerable to floor effects. An alternative approach is to restrict analyses to striatal subregions such as the anterior putamen or caudate, as these will be less likely to have reached floor effects.

Despite the recognized difficulties with DAT SBR as a quantitative outcome measure, it remains of major potential importance in the conduct of trials exploring disease‐modifying agents in PD.^15^ Although there is a surge in interest in recruiting patients at the earliest stages of PD, or even prior to the onset of motor manifestations, there will inevitably be a need to also assess the potential of candidate disease‐modifying interventions in the 10 million individuals who have already developed motor manifestations of PD but wish to avoid developing the falls, dementia, and swallowing issues associated with advanced PD.^16^

For these reasons, we sought to further explore how DAT SBR relates to clinical severity among patients with established PD, by performing analyses restricted to striatal subregions on both the most and least affected sides and considering the relationship between clinical subitem severity (lateralized bradykinesia, rigidity, tremor, and axial features) and these subregions. Our ultimate goal was to inform what should be the best approach for the analysis of DAT SBR as a disease‐modifying interventional trial outcome measure in patients with established PD.

We hypothesized the following:

1. In established PD, mean anterior putamen/caudate DAT SBR would be more strongly associated with MDS‐UPDRS, Part 3, score overall than mean posterior putamen DAT SBR.
2. The mean of bilateral whole striatal DAT SBR would be more strongly associated with axial features than lateralized striatal DAT SBR, but this relationship would lessen in advancing disease.
3. Lateralized anterior putamen and caudate DAT SBR would be more strongly associated with contralateral bradykinesia and rigidity than whole lateralized striatal DAT SBR in advancing disease.

## Patients and Methods

### Participants

Participants for this study were from a subgroup of recruits from the Exenatide PD2 and Exenatide PD3 trials who consented to undergo a DAT‐SPECT scan at trial baseline, prior to exposure to any investigational medications. Detailed trial recruitment criteria have previously been published.^17^, ^18^ Briefly, patients were aged between 25 and 80 years, had a clinical diagnosis of PD guided by Queen Square brain bank criteria, had a Hoehn and Yahr (H&Y) stage ≤2.5 in the *on* medication state, and had used dopaminergic treatment for at least 4 weeks. Participants who were suspected to have other causes for parkinsonism, with significant cognitive impairment (Montreal Cognitive Assessment <21) and/or concurrent severe depression, were excluded.

### Clinical Assessments

Demographic data included age, gender, and disease duration since diagnosis (DD). Motor characteristics were explored in the practically defined *off* medication state using the MDS‐UPDRS, Part 3. The *off* state was predefined as withholding all short‐acting conventional PD medications for at least 8 hours and all long‐acting conventional PD medications for at least 36 hours. Overall motor status was defined by the MDS‐UPDRS, Part 3, total score. Axial motor features were assessed using a composite Posture, Postural Instability and Gait (PIGD) score (items 3.9 + 3.10 + 3.11 + 3.12 + 3.13). Lateralized motor features were defined using right and left hemi body scores for tremor (items 3.15, 3.16, and 3.17), rigidity (item 3.3), and bradykinesia (items 3.4, 3.5, 3.6, 3.7, and 3.8).

### DAT Imaging Analysis

**FIG. 1:**
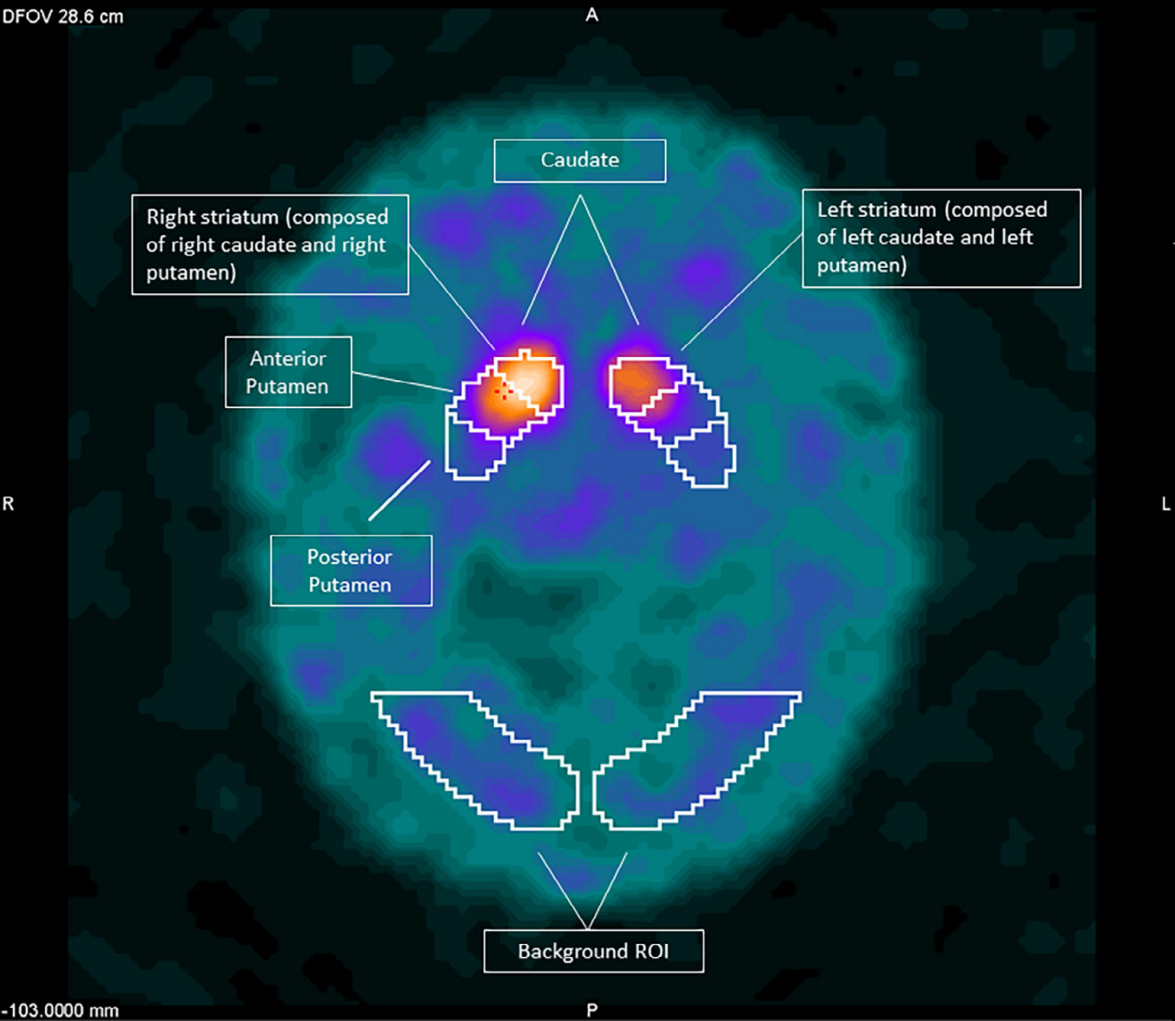
DaTQUANT SA volumes of interest (VOI) superimposed on a patient's automatically registered dopamine transporter single‐photon emission computed tomography (DAT‐SPECT) registered image.

DAT imaging was performed 3 hours after the intravenous injection of 185‐MBq Iodine‐123 Ioflupane using 1 of 2 GE Discovery 670 SPECT/CT scanners. No adjustments were made given the very similar performance of the 2 scanners. Data were acquired for 40 minutes and reconstructed using ordered subset expectation maximization iterative reconstruction with 2 iterations and 10 subsets with an image voxel size of 3 × 3 × 3 mm. After tomographic reconstruction, data analysis was performed using GE *DaTQUANT* software (DaTQUANT Stand alone (SA)). This is a fully automated quantification method, and its development methodology and workflow approach have previously been published.^19^ Briefly, *DaTQUANT* contains an image template based in MNI (Montreal Neurological Institute) space, which was defined on co‐registered T1 MR and DAT‐SPECT from the PPMI study. Within this template striatal volumes of interest covering the caudate and anterior and posterior putamen have been defined. The division of the anterior and posterior putamen was made arbitrarily during software development. When using the software, reconstructed DAT‐SPECT data from the subject are automatically spatially registered to this template, with the registered data visually assessed/adjusted by the operator (John Dickson) to ensure that the striatal contours fit the targets (Fig. 1). Using an occipital lobe region to represent nonspecific uptake in the brain, SBR values for the right and left overall striatum as well as the anterior and posterior putamen and caudate are obtained. SBR values are calculated by taking the count concentration in the region of interest and subtracting the count concentration in the nonspecific uptake volume before dividing this by the nonspecific count concentration.^20^ A mean score for the whole striatum and each subregion was determined by averaging the right and left scores for further analysis.

### Subgroups Explored

#### Disease Duration

Participants were divided into DD subgroups of ≤4 years and >4 years for subanalysis. No consensus on what a suitable disease duration cutoff would be currently exists for this approach. This cutoff was chosen based on a natural history study following newly diagnosed de novo patients demonstrating weaker DAT‐SPECT correlation with clinical markers at the 4‐year interval scan in contrast to the first and second years.^8^

#### Less‐Affected Side

We also explored analysis confined to the less‐affected side based on the SBR. No specific threshold for side‐to‐side difference was applied. Cases were included in this analysis if there was a difference between the sides, whereas cases not having any DAT SBR asymmetry were excluded.

### Statistical Analysis

Given nonnormally distributed data, medians and interquartile ranges were reported for continuous variables, whereas frequencies and percentages were reported for categorical variables. A Mann–Whitney *U*‐test was used for group comparison (disease duration ≤4 years vs. >4 years). χ^2^ test was used for comparing categorical data. Multivariate linear regression analysis was performed to investigate the relationship between mean and lateralized clinical scores (dependent variables) and different subregional (anterior putamen, posterior putamen, and caudate) hemispheric mean and lateralized SBR (independent variables). Age and gender were included in multivariate regression analysis due to their previously reported influence on clinical and DAT SBR outcomes.^21^, ^22^ The relationship of MDS‐UPDRS, Part 3, total scores and PIGD scores was assessed against mean bilateral DAT SBR. The relationship between lateralized clinical features (tremor, bradykinesia, and rigidity) was assessed against contralateral DAT SBR. Given the previous literature allowed clear hypotheses to be generated, we accepted a *P*‐value <0.05 to indicate statistical significance. All analyses were performed using *Stata*, version 17.0.

## Results

**FIG. 2:**
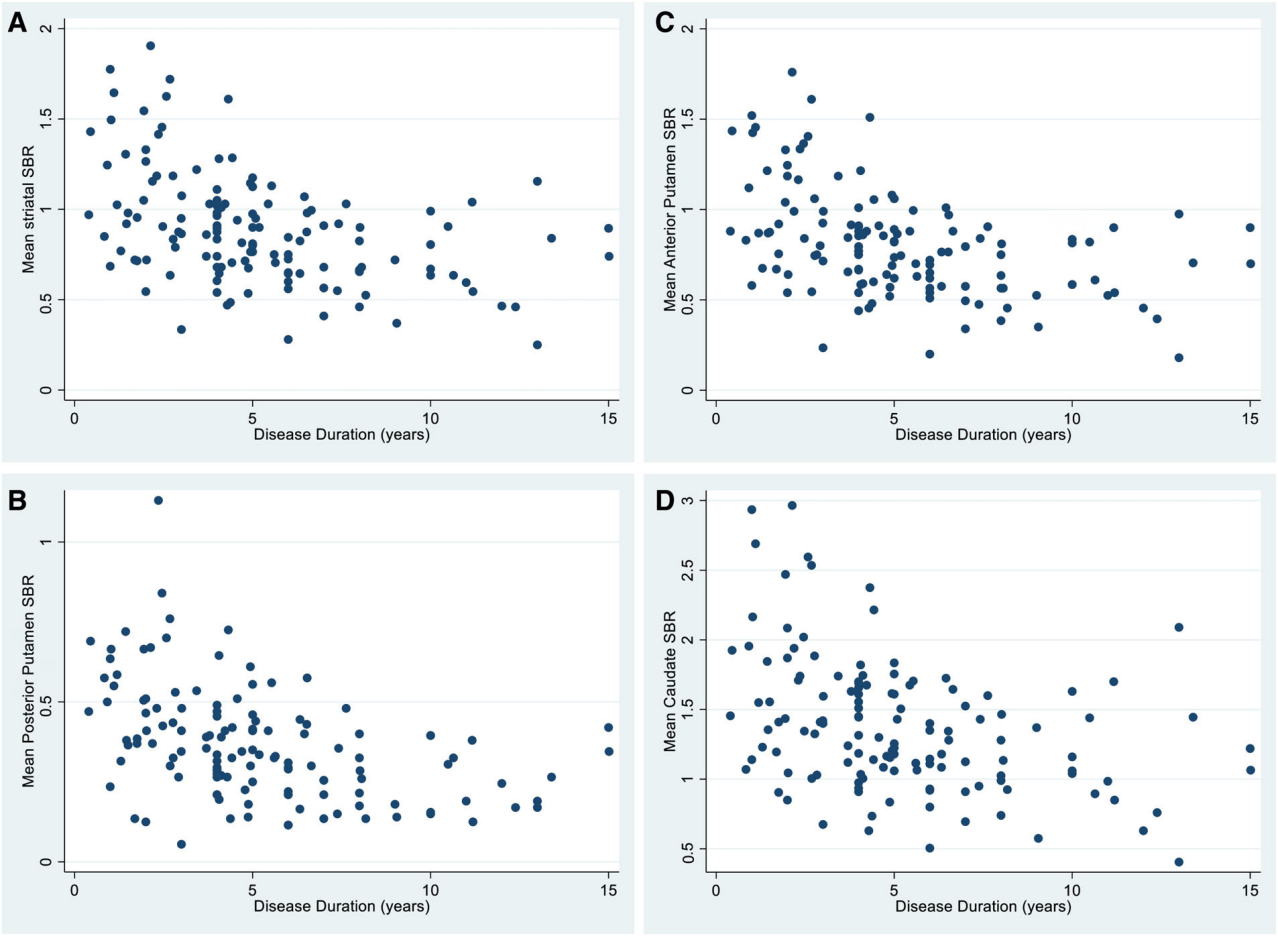
Distribution of mean hemispheric dopamine transporter striatal binding ratio (DAT SBR) by disease duration in (**A**) striatum, (**B**) posterior putamen, (**C**) anterior putamen, and (**D**) caudate.

One hundred thirty‐two patients with DAT‐SPECT assessments were included. All patients had abnormal imaging consistent with a parkinsonian disorder on visual inspection. Demographics and DAT SBR values are summarized in Table 1. The distribution of mean hemispheric DAT SBR of the overall striatum and striatal subregions by disease duration is shown in Figure 2. No significant age or gender differences were noted comparing subgroups with a disease duration ≤4 years and >4 years. Although there were (as expected) significant differences in the total MDS‐UPDRS, Part3, PIGD, and bradykinesia scores as well as DAT SBR in all striatal regions, there were no significant differences in rigidity or tremor scores between subgroups based on disease duration (Table 1).

**TABLE 1:** Cohort characteristics

Median (interquartile range)	Combined cohort (n = 132)	Disease duration <4 yr (n = 56)	Disease duration >4 yr (n = 76)	*P*‐value for subcohort comparisons
Age at baseline	60.09 (54.20–66.96)	61.15 (52.78–66.44)	59.89 (54.70–67.03)	0.7983
Disease duration	4.83 (2.83–7.00)	2.53 (1.72–3.75)	6.49 (5.00–9.00)	
Gender (M/F)	96/36	39/17	57/19	0.5470
MDS‐UPDRS, Part 3, total	32 (25–39.5)	30 (23–36)	34 (28–42.5)	**0.0170**
PIGD	3 (2–4)	3 (2–4)	4 (2.5–5)	**0.0026**
Tremor	2 (1–3)	2 (1–3)	2 (1–4)	0.4764
Bradykinesia	6 (4–8)	5 (4–7)	6 (4–8)	**0.0412**
Rigidity	2 (1–4)	2 (1–3)	2 (1–4)	0.3190
Mean SBR				
Whole striatum	0.86 (0.68–1.03)	0.97 (0.81–1.23)	0.76 (0.64–0.95)	**<0.0001**
Posterior putamen	0.35 (0.25–0.46)	0.41 (0.31–0.53)	0.30 (0.19–0.41)	**<0.0001**
Anterior putamen	0.78 (0.59–0.92)	0.88 (0.75–1.18)	0.70 (0.57–0.86)	**<0.0001**
Caudate	1.34 (1.05–1.65)	1.48 (1.21–1.86)	1.16 (0.99–1.49)	**0.0001**
Least affected SBR				
Whole striatum	0.96 (0.76–1.16)	1.03 (0.89–1.35)	0.83 (0.70–1.05)	**0.0001**
Posterior putamen	0.42 (0.31–0.56)	0.48 (0.39–0.67)	0.36 (0.25–0.47)	**<0.0001**
Anterior putamen	0.89 (0.70–1.05)	0.95 (0.87–1.32)	0.77 (0.64–0.97)	**<0.0001**
Caudate	1.46 (1.18–1.85)	1.60 (1.30–1.99)	1.31 (1.06–1.65)	**0.0007**

*Note*: Bold indicates significant values.

Abbreviations: MDS‐UPDRS, Movement Disorder Society Unified Parkinson's Disease Rating Scale, Part 3; PIGD, Posture, Postural Instability and Gait.

Table 2 summarizes the association between mean bilateral whole striatum and mean bilateral subregion SBR with the MDS‐UPDRS, Part 3, total score and the PIGD subscore. There was a nonsignificant trend between mean anterior putamen DAT SBR and MDS‐UPDRS, Part 3, but there was no significant relationship between overall bilateral striatal DAT SBR and MDS‐UPDRS, Part 3, scores. In contrast, there was a strong relationship between both whole bilateral striatum and regional DAT SBR for PIGD scores. Although the different disease duration subgroups are of smaller size, PIGD scores were associated only with the anterior putamen DAT SBR in the early disease subgroup.

**TABLE 2:** Relationship between nonlateralized motor scores and mean bilateral DAT SBR

Coefficient (SE), *P*‐value	Whole cohort MDS‐UPDRS, Part 3, total	MDS‐UPDRS, Part 3, total DD ≤ 4 years	MDS‐UPDRS, Part 3, total DD > 4 years	Whole cohort PIGD	PIGD DD ≤4 years	PIGD DD > 4 years
Overall striatum	−4.53 (3.13), 0.1504	−0.72 (4.34), 0.8692	−1.56 (5.23), 0.7663	**−2.04 (0.61), 0.0010**	−1.18 (0.73), 0.1125	−1.31 (1.02), 0.2048
Posterior putamen	−5.10 (5.32), 0.3391	2.74 (6.85), 0.6901	−4.60 (9.11), 0.6153	**−3.05 (1.04), 0.0039**	−1.57 (1.16), 0.1812	−2.63 (1.77), 0.1421
Anterior putamen	−5.87 (3.27), 0.0751	−1.80 (4.51), 0.6922	−3.85 (5.52), 0.4872	**−2.27 (0.63), 0.0005**	−1.48 (0.75), 0.0545	−1.61 (1.08), 0.1380
Caudate	−2.31 (2.08), 0.2673	−0.22 (2.93), 0.9400	0.38 (3.31), 0.9094	**−1.18 (0.40), 0.0041**	−0.59 (0.50), 0.2390	−0.58 (0.65), 0.3764

*Note*: Bold indicates significant values.

Abbreviations: DAT SBR, dopamine transporter striatal binding ratio; SE, standard error; MDS‐UPDRS, Movement Disorder Society Unified Parkinson's Disease Rating Scale; DD, disease duration since diagnosis; PIGD, Posture, Postural Instability and Gait.

Table 3 summarizes the associations between lateralized whole striatum and lateralized striatal subregion DAT SBR and different lateralized motor characteristics. Bradykinesia scores were significantly associated with DAT SBR in all subregions, whereas tremor and rigidity scores were associated only with more anterior striatal structures. Bradykinesia remained strongly associated with anterior striatal DAT SBR values even with advancing disease, whereas tremor and rigidity were no longer associated.

**TABLE 3:** Association between specific motor scores and lateralized SBR

	Coefficient (SE), *P*‐value					
	Including most and least affected sides			Including least affected side only		
	Combined trial cohorts	Disease duration ≤4 years	Disease duration >4 years	Combined trial cohorts	Disease duration ≤4 years	Disease duration >4 years
Contralateral tremor						
Whole striatum	**−1.31 (0.47), 0.0055**	**−1.67 (0.67), 0.0139**	−0.96 (0.77), 0.2168	−0.33 (0.52), 0.5204	−0.23 (0.71), 0.7474	0.01 (0.89), 0.9867
Posterior putamen	−1.18 (0.73), 0.1043	−1.55 (0.95), 0.1045	−0.42 (1.25), 0.7367	−0.32 (0.96), 0.7340	0.46 (1.17), 0.6983	0.34 (1.84), 0.8526
Anterior putamen	**−1.46 (0.48), 0.0027**	**−1.84 (0.67), 0.0070**	−1.10 (0.80), 0.1724	−0.54 (0.55), 0.3275	−0.21 (0.74), 0.7777	−0.36 (0.97), 0.7164
Caudate	**−0.83 (0.31), 0.0097**	**−1.03 (0.47), 0.0283**	−0.60 (0.49), 0.2288	−0.14 (0.32), 0.6705	−0.11 (0.50), 0.8272	0.33 (0.48), 0.9442
Contralateral bradykinesia						
Whole striatum	**−2.35 (0.57), <0.0001**	−1.37 (0.86), 0.1137	**−2.72 (0.88), 0.0023**	**−1.39 (0.70), 0.0482**	−0.22 (1.04), 0.8325	−1.66 (1.13), 0.1457
Posterior putamen	**−2.44 (0.89), 0.0064**	−1.77 (1.20), 0.1434	−2.24 (1.45), 0.1232	−2.14 (1.24), 0.0874	0.31 (1.54), 0.8409	−1.43 (2.21), 0.5217
Anterior putamen	**−2.71 (0.58), <0.0001**	**−1.71 (0.86), 0.0496**	**−3.33 (0.90), 0.0003**	**−1.65 (0.74), 0.0277**	−0.54 (1.10), 0.6245	−2.02 (1.22), 0.1025
Caudate	**−1.36 (0.38), 0.0005**	−0.70 (0.60), 0.2475	**−1.40 (0.57), 0.0144**	−0.90 (0.48), 0.0641	0.24 (0.73), 0.7397	−1.23 (0.72), 0.0919
Contralateral rigidity						
Whole striatum	−0.54 (0.29), 0.0642	−0.71 (0.42), 0.0949	−0.44 (0.48), 0.3637	−0.08 (0.38), 0.8399	−0.43 (0.59), 0.4721	0.21 (0.61), 0.7375
Posterior putamen	−0.24 (0.45), 0.5912	−0.38 (0.59), 0.5231	0.13 (0.77), 0.8624	0.08 (0.63), 0.9056	0.28 (0.80), 0.7242	0.90 (1.19), 0.4542
Anterior putamen	**−0.69 (0.30), 0.0214**	−0.73 (0.42), 0.0870	−0.75 (0.49), 0.1290	0.02 (0.39), 0.9662	−0.34 (0.58), 0.5611	0.37 (0.65), 0.5723
Caudate	−0.32 (0.20), 0.1016	−0.53 (0.29), 0.0730	−0.17 (0.31), 0.5748	0.15 (0.25), 0.5517	−0.19 (0.39), 0.6264	0.41 (0.37), 0.2809

*Note*: Bold indicates significant values.

Abbreviations: SBR, striatal binding ratio; SE, standard error.

When analysis was restricted to the less‐affected side, 6 patients did not have anterior putamen asymmetry, 2 did not have posterior putamen asymmetry, and 3 did not have caudate asymmetry and were therefore excluded from respective regression analysis. Similar relationships were observed, with the anterior putamen being more consistently associated with bradykinesia than the posterior putamen again likely due to the high variance (standard error) seen in the posterior putamen DAT SBR results. However, the smaller sample size led to fewer significant associations when the only least affected sides were included, particularly in the cohort split based on disease duration.

## Discussion

In this study we aimed to evaluate the relationships between the severity of dopaminergic denervation in the whole striatum and its subregions and the severity of lateralized clinical motor features, to help inform on how DAT SBR might be optimally analyzed as an outcome measure in disease‐modifying trials. In this cross‐sectional dataset, we found as expected that MDS‐UPDRS, Part 3, scores were higher comparing early versus more advanced disease, and DAT SBR scores were lower when considering the mean score across the whole striatum or in all striatal subregions. There was, however, no significant relationship between MDS‐UPDRS, Part 3, severity and mean bilateral DAT SBR. In contrast, the clinical change (in the *off* medication state) was most consistently driven by PIGD features becoming more severe, and these more closely mirror the severity of mean bilateral DAT SBR loss than bradykinesia, rigidity, or tremor. PIGD items undoubtedly have a bilateral contribution, therefore the strong association with mean DAT SBR; however, when we restricted focus to the DAT SBR in lateralized anterior putamen and lateralized scores of clinical subitems, we found the most statistically significant relationships were between lateralized anterior putamen DAT SBR scores and the contralateral bradykinesia score. This was maintained even beyond a disease duration of 4 years.

Our finding that tremor and rigidity scores did not change significantly between our earlier disease patients and our more established patients is of interest. This is likely explicable in view of higher variability of these clinical items between patients, even in early disease. Lack of consistent progression in these items may mean that clinical progression may be somewhat diluted when considering overall MDS‐UPDRS, Part 3, scores rather than exploring specific subitems. Tremor has differentially lateralized rest, postural, and kinetic aspects, and the underlying dopaminergic and nondopaminergic bases of these tremor elements vary^23^, ^24^ and can change over the disease course,^23^, ^25^ perhaps also under the influence of long‐duration effects of dopaminergic replacement therapies.

Nevertheless, MDS‐UPDRS, Part 3, scores in the *off* medication state do obviously change over time, and in our cohort, a significant contribution to this appears to be the increase in the PIGD subitems in this scale. The change in PIGD subitems is associated with changes in all DAT SBR subregions but particularly the anterior putamen and more so in earlier disease. Although PIGD scores may be a useful clinical measurement of change related to striatal denervation in early disease, with more advanced disease, the relationship is again likely diluted due to additional contributions from extra‐striatal denervation. Also, a near‐maximal decrease in mean striatal DAT SBR is noted ~4 to 5 years post‐onset in our cohort (Fig. 2). This is most prominently observed in the posterior and anterior putamen subregions. This horizontal asymptote in the SBR in the later disease duration stage will partly contribute to the less‐significant relationship noted.

The most consistent relationship between lateralized DAT SBR and lateralized motor characteristics was noted in the anterior putamen even across the patient subgroups studied (despite the inevitably smaller sample sizes). This may partly reflect that denervation in the posterior putamen has reached a floor effect very early in the course of disease progression. The division of the anterior and posterior putamen during the image analysis is arbitrary, and specific to this particular software that was used although is consistently applied. As a consequence, the true extent of the area showing this relationship is unknown, but the finding is still valuable in terms of its approximate regionality.

Restricting analysis to the less‐affected side did not profoundly influence these findings. In addition, there are challenges with the registration of the posterior putamen region to the template given that it has low or no signal, thus further impacting on reliability of measurement in this region. Denervation in the caudate may be less closely related to the motor characteristics we explored (lower‐association coefficients in our study and previous studies demonstrating stronger associations with cognitive performance^26^, ^27^). Loss of DAT SBR in the caudate tends to occur later as suggested by its significant association with bradykinesia only being noted in later disease.

DAT SBR reflects the loss of functioning dopaminergic terminals in the striatum. Ratios therefore correlate best with clinical deficits that are related to the dopamine transporter system.^5^ Our findings are in line with this and broadly mirror previous studies,^5^, ^28^, ^29^, ^30^, ^31^ particularly with contralateral bradykinesia and, to a lesser extent, with rigidity. If considering an intervention that is targeted to rescue degenerating dopaminergic neurons, DAT SBR may therefore be a more sensitive measure of change than crude clinical evaluations of bradykinesia. This does not of course capture nondopaminergic degeneration, and it may be less likely that any disease‐modifying intervention would be restricted to dopaminergic terminals alone, but DAT SBR may nevertheless potentially still be a more useful, objective, and sensitive measure of target engagement and potential efficacy even among individuals with >4 years of disease. Radionuclide imaging of the serotonergic, noradrenergic, and cholinergic systems demonstrates associations with nonmotor PD pathophysiology.^32^ These approaches may be of particular importance in later disease stages where cognitive impairment and gait abnormalities become more prominent though the utility of these markers in tracking progression will need more detailed evaluation.^33^, ^34^, ^35^

Disease duration is a fundamental issue in the design of clinical trials in PD, with increasing attention toward intervening early or even in premotor PD.^16^ Nevertheless, any early signal of success will likely require replication/confirmation among people with established motor PD, which will need a sensitive measure to detect efficacy. The incorporation of DAT SBR as a potential outcome measure in trials of disease‐modifying interventions will therefore likely remain of great interest.^5^ Correlation between DAT SBR and motor deficits becomes weaker with advancing disease duration from diagnosis.^8^ This is in part related to floor effects, with 1 pathological study suggesting a virtual absence of fibers in the dorsal striatum 4 years after diagnosis.^36^ These findings are broadly in line with the disease duration differences we noted though the larger variances as demonstrated by the large standard errors we report in the regression analysis of the longer‐duration group may also be partly explained by smaller sample sizes.^37^ The reduced strength of association noted does not however entirely exclude the use of DAT SBR subregions in patients with longer disease duration, particularly given our findings regarding contralateral bradykinesia and anterior putaminal DAT uptake.

We have shown that lateralized DAT SBR usefully predicts the severity of some motor characteristics in our cohort of PD patients. If applied as an outcome measure in a disease‐modifying trial, there are several potential methods of analyzing DAT SBR data. Changes in each lateralized anterior putamen DAT SBR could be analyzed based on active treatment/placebo, that is, 2 data points per participant in an early‐phase trial as an early sensitive signal, whereas in a later phase 3 trial, the same lateralized anterior putamen DAT SBRs might contribute to a composite trial endpoint that also encompasses clinical or patient‐reported measures. The analysis of the least affected side was designed to disentangle floor effects. Although consistent with the findings using both DAT SBRs, isolating analysis to the lateralized region that is least affected did not provide additional value in our cohort, and reducing the amount of data by 50% might result in a negative impact on power for any given trial sample size calculation.

Although our total cohort of patients is relatively large, subgrouping based on disease duration may have impacted on our ability to detect significant associations. We had very few participants with young‐onset PD to address the impact of heterogeneity arising from this subgroup. Our overarching goal was to explore relationships using different DAT SBR analysis approaches to inform best approaches for demonstrating disease modification. We acknowledge that the focus of this work was on patients with mild to moderate disease defined largely by a H&Y score of ≤2.5 and the absence of significant cognitive impairment. This excludes a large proportion of PD patients who fall into preclinical, de novo, and severe disease stages of the disease, thus potentially limiting the generalizability of our findings though our study cohort is a target group for a range of important therapeutic trials.^16^ Our analysis used cross‐sectional data and relied on accurate disease duration data, which might be subject to recall bias or lack of recognition of motor PD in its early stages. Furthermore although we adjust for age and gender in our association analysis, we acknowledge that several other biological and technical factors can influence the relationship between DAT SBR and clinical severity and have not been considered in our modeling.^38^ In addition, we analyzed the association between *off* state motor scores and DAT SBR in the *on* state. Impact on striatal DAT levels from dopaminergic medication is however difficult to adjust for considering the varying impact of levodopa and dopamine agonists, short‐ and long‐acting agents, as well as differential impacts based on disease duration.^39^, ^40^ Our work has explored 1 presynaptic ligand approach, and we must acknowledge that several other approaches with relative benefits exist and may ultimately be superior for demonstrating disease modification.^15^ Although a disease‐modifying intervention may have different or overlapping effects on both motor and nonmotor features of PD, we propose that future analysis might consider limiting DAT SBR measurement to the anterior putamen for demonstrating disease‐modifying effects of interventions in patients with clinically established PD. Whether DAT SBR proves to be a useful outcome measure in trials of candidate disease‐modifying interventions will depend on the identification of a successful treatment. Once proven, it may become a useful means of shortening the length of follow‐up needed to confirm/refute effects.

## Author Roles

(1) Research project: A. Conception, B. Organization, C. Execution; (2) Statistical analysis: A. Design, B. Execution, C. Review and critique; (3) Manuscript preparation: A. Writing of the first draft, B. Review and critique.

N.V.: 1A, 1B, 1C, 2A, 2B, 2C, 3A, 3B

C.G.: 1B, 1C, 2C, 3B

D.A.: 1B, 1C, 2C, 3B

A.K.: 1B, 1C, 3B

G.A.: 1B, 1C, 3B

R.M.: 1B, 1C, 3B

K.C.: 1B, 1C, 3B

S.S.: 1B, 1C, 3B

K.M.: 1B, 1C, 3B

K.R.C.: 1C, 3B

V.L.: 1A, 1B, 1C, 3B

J.D.: 1A, 1B, 1C, 2C, 3B

T.F.: 1A, 1B, 1C, 2A, 2B, 2C, 3A, 3B

## Disclosures

**Ethical Compliance Statement:** The Exenatide PD3 trial received REC (initial date of approval: October 15, 2019, REC reference no.: 19/SC/0447) and other regulatory approvals (EudraCT 2018–003028‐35). The Exenatide PD2 trial received REC (initial date of approval: November 11, 2013, REC reference no.: 13/LO/1536) and other regulatory approvals (EudraCT 2013–003363‐64). Informed written consent was obtained from all patients recruited for the clinical trials, and separate written consent was also obtained for those participating in the DAT‐SPECT substudy. We confirm that we have read the journal's position on issues involved in ethical publication and affirm that this work is consistent with those guidelines.

**Funding Sources and Conflicts of Interest:** Exenatide PD3 was funded by the National Institute for Health Research (NIHR) Efficacy and Mechanism Evaluation (EME) Programme, an MRC and NIHR partnership (project number: 16/167/19), and Cure Parkinson's. Exenatide PD2 was funded by the Michael J. Fox foundation. Nirosen Vijiaratnam's research time and position is funded by the Janet Owens Legacy Fund. The authors declare that there are no conflicts of interest relevant to this work.

**Financial Disclosures for the Previous 12 Months:** Nirosen Vijiaratnam has served on the advisory boards of Bial and AbbVie and received honoraria from Bial. He is supported by a Janet Owens Research Fellowship, awarded to Thomas Foltynie. Christine Girges reports no financial disclosures. Dilan Athauda reports no financial disclosures. Alexa King reports no financial disclosures. Grace Auld reports no financial disclosures. Rachel McComish reports no financial disclosures. Kashfia Chowdhury reports no financial disclosures. Simon Skene reports no financial disclosures. Kate Maclagan reports no financial disclosures. Kallol Ray Chaudhuri is a study investigator and has served as an advisory board member for AbbVie, UCB, GKC, Bial, Cynapsus, Lobsor, Stada, Medtronic, Zambon, Profile, Sunovion, Roche, Theravance, Scion, Britannia, Acadia, and 4D. He received honoraria for lectures from AbbVie, Britannia, UCB, Zambon, Novartis, Boehringer Ingelheim, Bial, Kyowa Kirin, and SK Pharma. He has received grants (investigator initiated) from Britannia Pharmaceuticals, AbbVie, UCB, GKC, and Bial, and academic grants from EU, IMI EU, Horizon 2020, Parkinson's UK, NIHR, PDNMG, EU (Horizon 2020), Kirby Laing Foundation, NPF, MRC, and Wellcome Trust. He receives royalties from the Oxford University Press and holds intellectual property rights for the King's Parkinson's Pain Scale and Parkinson's Disease Sleep Scale. Vincenzo Libri has served on the advisory boards of Nova Pharmaceuticals, Amazentis Life Sciences, and Biogen, and on Independent Data Monitoring boards for VICO Therapeutics, QurAlis, and Cyclo Therapeutics. He has received grants from the National Institute of Health Research and United Therapeutics. John Dickson reports no financial disclosures. Thomas Foltynie has served on the advisory boards of Bial, AbbVie, Nodthera, Treefrog, Novonordisk, BlueRock, and Bayer. He has received grants from the National Institute of Health Research, the Michael J. Fox Foundation, the Edmond J. Safra Foundation, the John Black Charitable Foundation, Cure Parkinson's Trust, Innovate UK, the Van Andel Research Institute, and Defeat MSA. He has received honoraria for talks sponsored by Bial, Bayer, Boston Scientific, and Novonordisk.

## Data Availability

Data from this study are available on request from the authors.
